# Author Correction: Asymmetric requirement of Dpp/BMP morphogen dispersal in the *Drosophila* wing disc

**DOI:** 10.1038/s41467-021-27680-z

**Published:** 2022-01-14

**Authors:** Shinya Matsuda, Jonas V. Schaefer, Yusuke Mii, Yutaro Hori, Dimitri Bieli, Masanori Taira, Andreas Plückthun, Markus Affolter

**Affiliations:** 1 Biozentrum, University of Basel, Basel, Switzerland; 2 Department of Biochemistry, University of Zurich, Zurich, Switzerland; 3 National Institute for Basic Biology and Exploratory Research Center on Life and Living Systems (ExCELLS), National Institutes of Natural Sciences, Okazaki, Aichi Japan; 4 JST PRESTO, Kawaguchi, Saitama Japan; 5 Institute for Quantitative Biosciences, The University of Tokyo, Tokyo, Japan; 6 Department of Biological Sciences, Graduate School of Science, The University of Tokyo, Tokyo, Japan; 7 Present Address: Department of Biological Sciences, Faculty of Science and Engineering, Chuo University, Tokyo, Japan

**Keywords:** Genetic engineering, Synthetic biology, Morphogen signalling, Limb development, Pattern formation

Correction to: *Nature Communications* 10.1038/s41467-021-26726-6, published online 8 November 2021.

In this article the grant number 310030_192659 relating to SNSF for Markus Affolter was omitted. The original article has been corrected.

