# Supplementary material for: Asymmetric requirement of Dpp/BMP morphogen dispersal in the Drosophila wing disc
Source: Nat Commun. 2021 Nov 8;12:6435. doi: 10.1038/s41467-021-26726-6 (PMC8576045; doi:10.1038/s41467-021-26726-6)
Supplement: Supplementary file 1 — Supplementary Information [file 41467_2021_26726_MOESM1_ESM.pdf]

# SUPPLEMENTARY INFORMATION

**Supplementary Figure 1-13**

**Supplementary Table 1**: Genotypes by figures

**Supplementary Table 2**: Primers used in this study

## SUPPLEMENTARY FIGURES

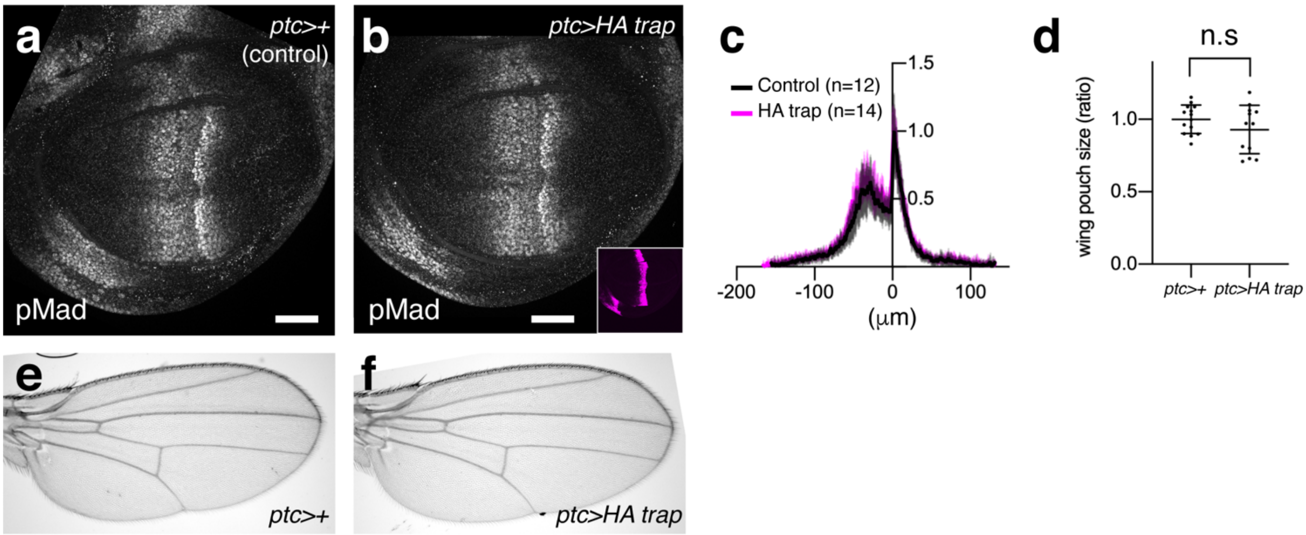


**Supplementary Fig 1. Expression of HA trap using *ptc*-Gal4 did not affect Dpp signaling or patterning and growth of the adult wing in the absence of a HA-tagged protein.**
**a-b**, pMad staining of *ptc*>+ wing disc (control) (**a**), and *ptc>HA trap* wing disc (**b**). Scale bar 50 μm. **c**, Average fluorescence intensity profile of α-pMad staining of (**a-b**). *ptc*>+ wing disc (control) (*n*=12), and *ptc>HA trap* wing disc (*n*=14). Data are presented as mean+/-SD. **d**, Comparison of wing pouch size of (**a-b**). *ptc*>+ wing disc (control) (*n*=14) and *ptc>HA trap* wing disc (*n*=12). Data are presented as mean+/-SD. Two-sided unpaired Student's *t*-test with unequal variance was used (*p*=0.211). (n.s; not significant). **e-f**, Adult wing of *ptc*>+ (**e**), and *ptc>HA trap* (**f**).

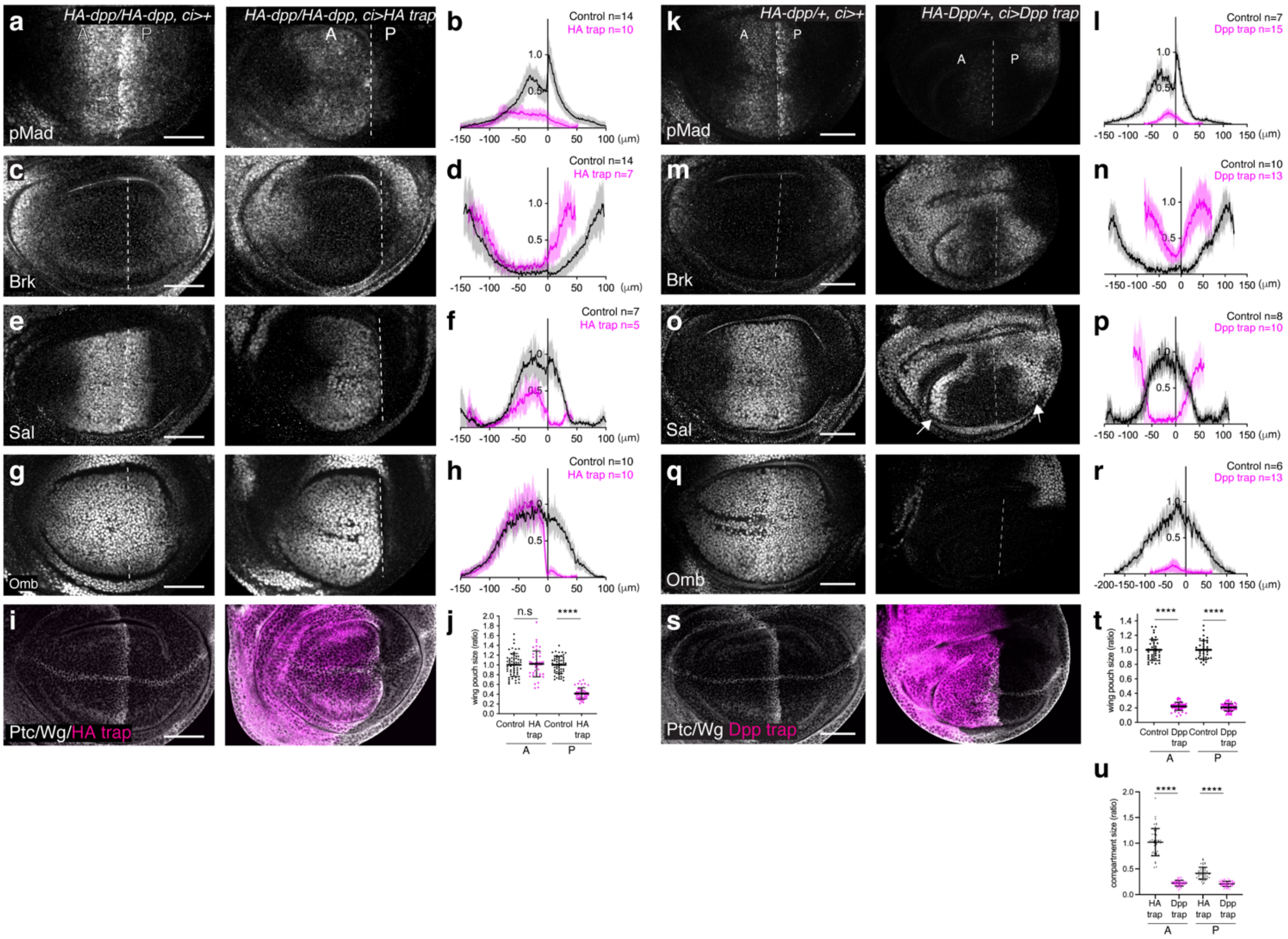


**Supplementary Fig 2. Patterning and growth defects by HA trap and Dpp trap expression using *ci-Gal4***

**a–j**, Patterning and growth defects by HA trap. (**a**, **c**, **e**, **g**, **i**) α-pMad (**a**), α-Brk (**c**), α-Sal (**e**), α-Omb (**g**), α-Ptc/Wg staining and HA trap (mCherry) (**i**) of control *HA-dpp/HA-dpp, ci>+* (left) and *HA-dpp/HA-dpp, ci>HA* trap (right). (**b**, **d**, **f**, **h**) Average fluorescence intensity profile of (**a**, **c**, **e**, **g**) respectively. Data are presented as mean+/-SD. (**j**) Comparison of compartment size of *HA-dpp/HA-dpp, ci>+* wing pouch (control) (*n*=56) and *HA-dpp/HA-dpp, ci>HA* trap wing pouch (*n*=47). Data are presented as mean+/-SD. Two-sided Mann-Whitney test was used for comparison of the A compartment size ($p$=0.8355). (n.s; not significant) Two-sided unpaired Student's *t*-test with unequal variance was used for comparison of the P compartment size ($p$<0.0001). (****$p$<0.0001). **k-t**, Patterning and growth defects by Dpp trap. (**k**, **m**, **o**, **q**, **s**) α-pMad (**k**), α-Brk (**m**), α-Sal (**o**), α-Omb (**q**), α-Ptc/Wg staining and HA trap (mCherry) (**s**) of control *HA-dpp/+, ci>+* (left) and *HA-dpp/+, ci>Dpp* trap (right). (**l**, **n**, **p**, **r**) Average fluorescence intensity profile of (**k**, **m**, **o**, **q**) respectively. Data are presented as mean+/-SD. **t**, Comparison of each compartment size of *HA-dpp/+, ci>+* wing pouch (control) (*n*=37) and *HA-dpp/+, ci>Dpp* trap wing pouch (*n*=53). Data are presented as mean+/-SD. Two-sided unpaired Student's *t*-test with unequal variance was used for comparison of the A compartment size ($p$<0.0001) and for comparison of the P compartment size ($p$<0.0001). (****$p$<0.0001). **u**, Comparison of normalized compartment size of wing pouch upon HA trap (*n*=47) and Dpp trap (*n*=53) expression using *ci-Gal4* (the same data set from Supplementary Fig. 2j and Supplementary Fig. 2t). Data are presented as mean+/-SD. Two-sided Mann-Whitney test was used for comparison of the A compartment size ($p$<0.0001). Two-sided unpaired Student's *t*-test with unequal variance was used for comparison of the P compartment size ($p$<0.0001). Dashed white lines mark the A-P compartment border. Scale bar 50 µm.

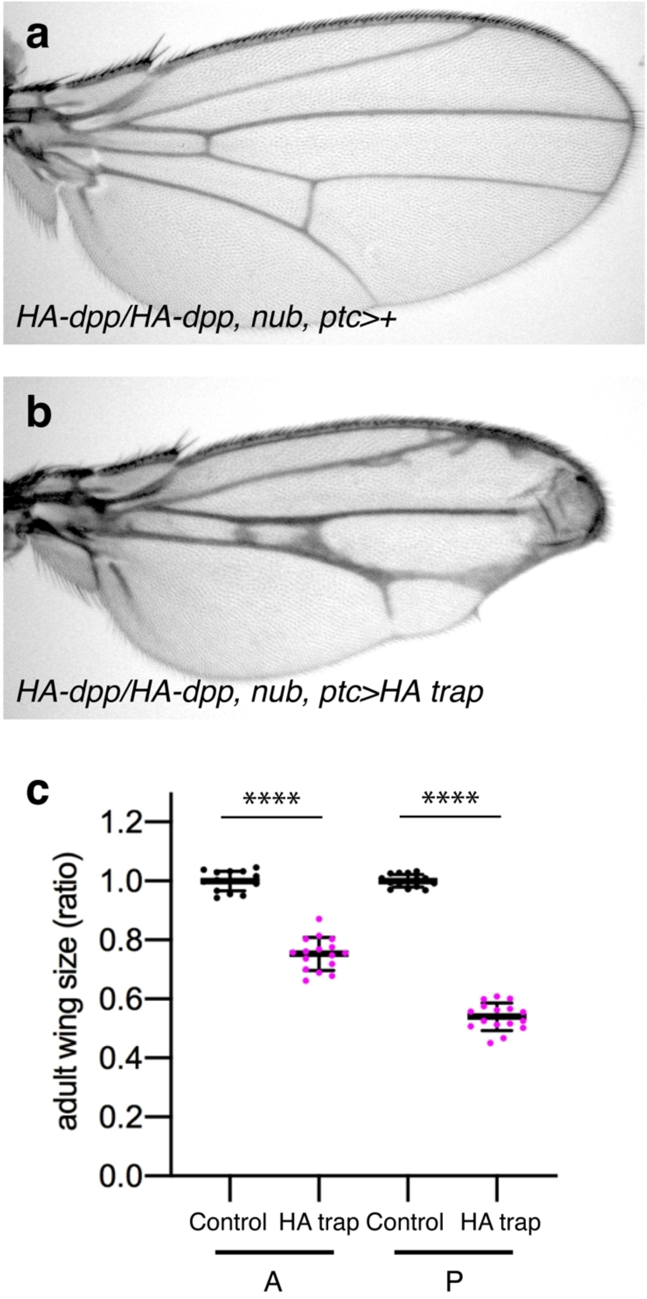


**Supplementary Fig 3. Patterning and growth defects by concomitant HA trap expression using *ptc-Gal4* and *nub-Gal4***

**a**-**b**, Adult wing of control *HA-dpp/HA-dpp, nub, ptc>+* (**a**), and *HA-dpp/HA-dpp, nub, ptc>HA trap* (**b**). **c**, Comparison of compartment size of (**a**-**b**). *HA-dpp/HA-dpp, nub, ptc>+* adult wing ($n$=15) and *HA-dpp/HA-dpp, nub, ptc>HA trap* adult wing ($n$=16). Data are presented as mean+/-SD. Two-sided unpaired Student's $t$-test with unequal variance was used for comparison of the A compartment size ($p$<0.0001) and for comparison of the P compartment size ($p$<0.0001). (****$p$<0.0001). Note that patterning and growth defects were not enhanced by concomitant HA trap expression using *ptc-Gal4* and *nub-Gal4*.

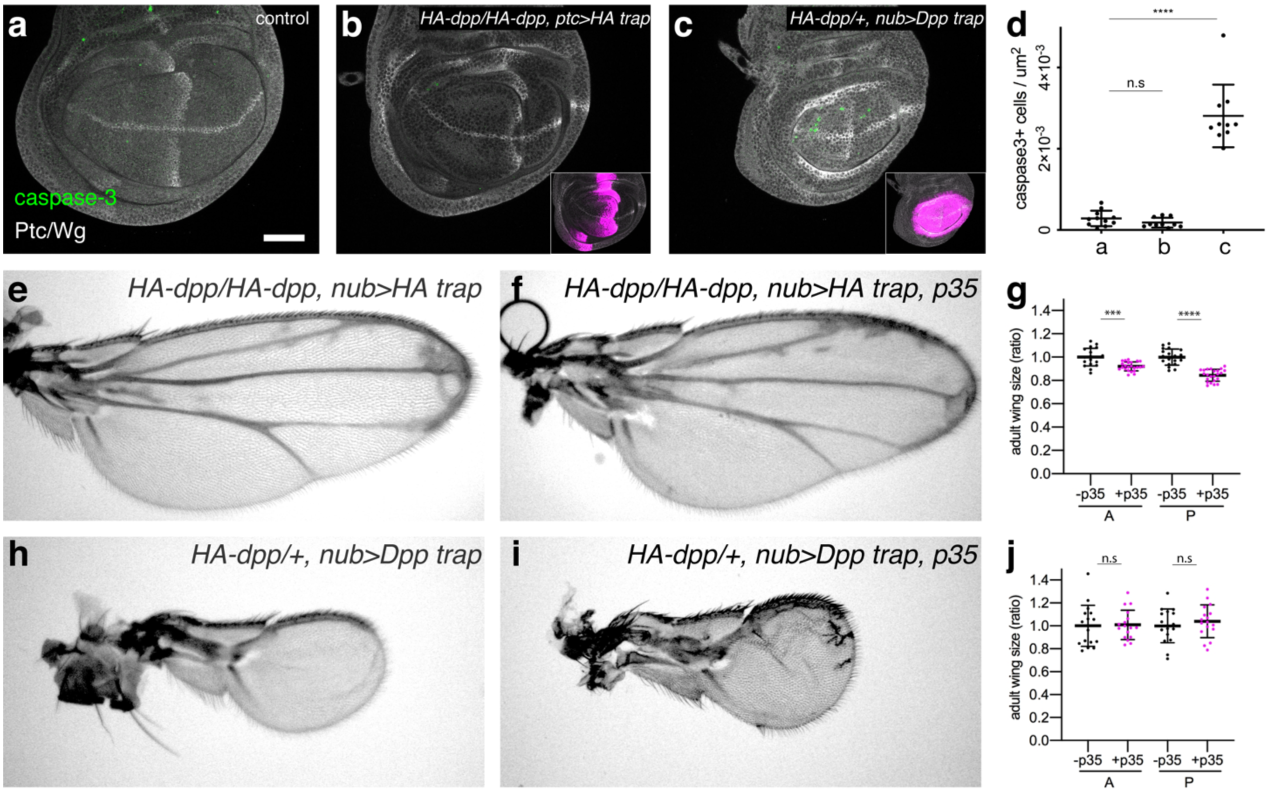


**Supplementary Fig 4. Blocking cell death does not rescue growth defects caused by HA trap or Dpp trap**

**a-d**, α-Caspase-3 and α-Ptc/Wg staining of control wing disc (**a**), *HA-dpp/HA-dpp, ptc>HA trap* wing disc (**b**), and *HA-dpp/+, nub>Dpp trap* wing disc (**c**). The insets show HA trap or Dpp trap (mCherry) expression. Scale bar 50 µm. Note that wing discs where HA trap was expressed using *ptc*-Gal4 and wing discs where Dpp trap was expressed using *nub*-Gal4 were analyzed since each condition showed the most severe phenotypes among Gal4 lines used. (**d**) Comparison of the number of α-Caspase-3 positive cells of (**a-c**). Control wing disc (**a**, $n$=11), *HA-dpp/HA-dpp, ptc>HA trap* wing disc (**b**, $n$=10), and *HA-dpp/+, nub>Dpp trap* wing disc (**c**, $n$=10). Data are presented as mean+/-SD. Two-sided unpaired Student's $t$-test with unequal variance was used for comparison between (**a**) and (**b**) ($p$=0.1541). (n.s; not significant). Two-sided Mann-Whitney test was used for comparison between (**a**) and (**c**) ($p<0.0001$). (****$p<0.0001$). **e-f**, Adult wing of *HA-dpp/HA-dpp, nub>HA trap* (control) (**e**) and *HA-dpp/HA-dpp, nub>HA trap, p35* (**f**). **g**, Comparison of compartment size of (**e-f**). *HA-dpp/HA-dpp, nub>HA trap* adult wing ($n$=19) and *HA-dpp/HA-dpp, nub>HA trap, p35* adult wing ($n$=23). Data are presented as mean+/-SD. Two-sided unpaired Student's $t$-test with unequal variance was used for comparison of the A compartment size ($p$=0.0003) and for comparison of the P compartment size ($p<0.0001$). (***$p<0.001$, ****$p<0.0001$). **h-i**, Adult wing of *HA-Dpp/+, nub>Dpp trap* (control) (**h**) and *HA-dpp/+, nub>Dpp trap, p35* (**i**). **j**, Comparison of compartment size of (**h-i**). *HA-Dpp/+, nub>Dpp trap* adult wing ($n$=17) and *HA-dpp/+, nub>Dpp trap, p35* adult wing ($n$=17). Data are presented as mean+/-SD. Two-sided unpaired Student's $t$-test with unequal variance was used for comparison of the A compartment size ($p$=0.8750) and for comparison of the P compartment size ($p$=0.4260). (n.s; not significant).

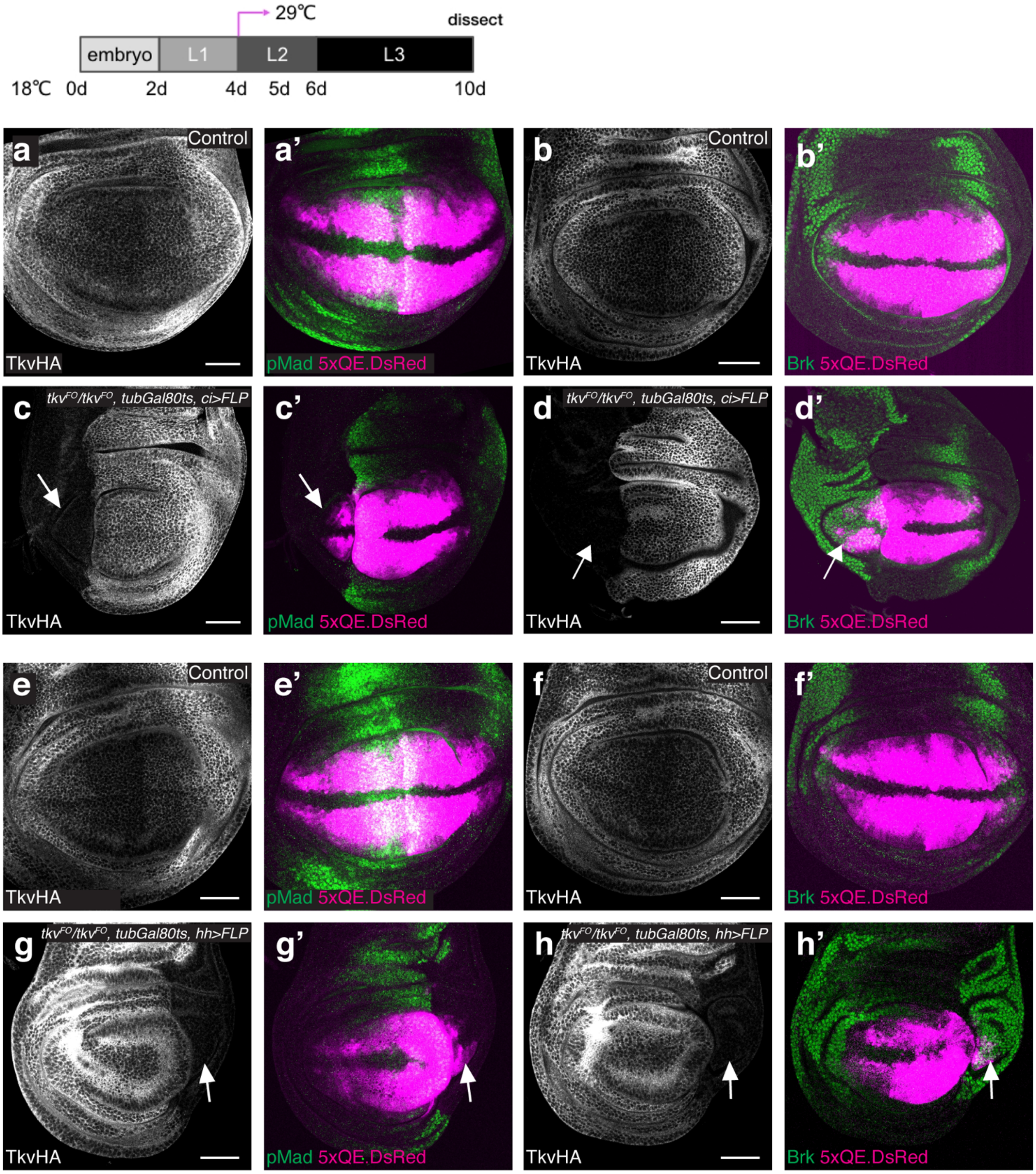


**Supplementary Fig 5. *5xQE.DsRed* remains expressed in each compartment where *tkv* is genetically removed from the beginning of second instar stage.**

**a-d**, α-HA (TkvHA$^{FO}$) staining (**a-d**) and α-Brk staining and 5xQE.DsRed expression (**a'-d'**) of control wing disc (**a**, **b**) and *5xQE.DsRed, tkvHA$^{FO}$/tkvHA$^{FO}$, tubGal80ts, ci>UAS-FLP* (**c**, **d**). **e-h**, α-HA (TkvHA$^{FO}$) staining (**e-h**) and α-Brk staining and 5xQE.DsRed expression (**e'-h'**) of control wing disc (**e**, **f**) and *5xQE.DsRed, tkvHA$^{FO}$/tkvHA$^{FO}$, tubGal80ts, hh>UAS-FLP* (**g**, **h**). Crosses were shifted from 18°C to 29°C at 4 day AEL (early second instar). Arrows indicate 5xQE.DsRed expression in the compartment where *tkv* is genetically removed. Scale bar 50 µm.

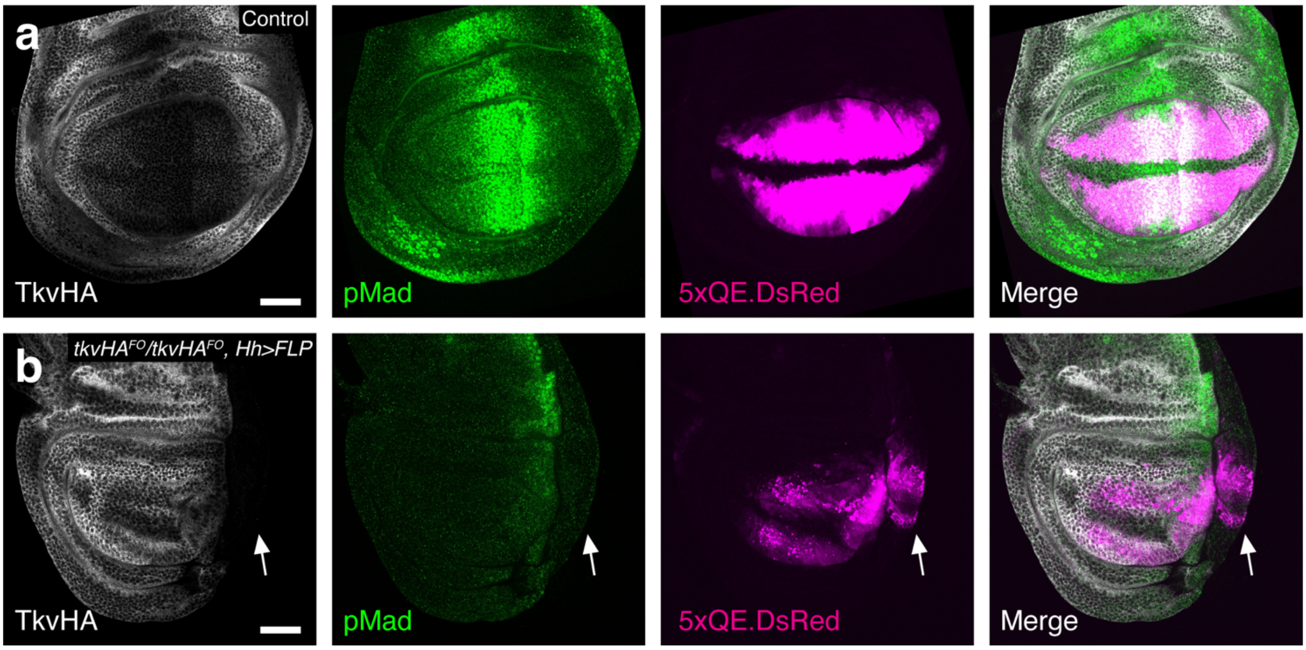


**Supplementary Fig 6. A part of posterior wing pouch can grow without *tkv*.**
**a**-**b**, α-HA (TkvHA$^{FO}$), α-pMad, 5xQE.DsRed, and merge of control wing disc (**a**), and wing disc where *tkv* is genetically removed from the entire P compartment using *Hh*-Gal4 (**b**). Upon removal of *tkv* from the P compartment, the 5xQE.DsRed reporter remained expressed in the P compartment (arrow) despite complete loss of pMad signal and severe growth defects in the P compartment. Note that anterior pMad signal was also affected probably because Hh target *dpp* expression is affected by the reduced number of Hh producing posterior cells. Scale bar 50 μm.

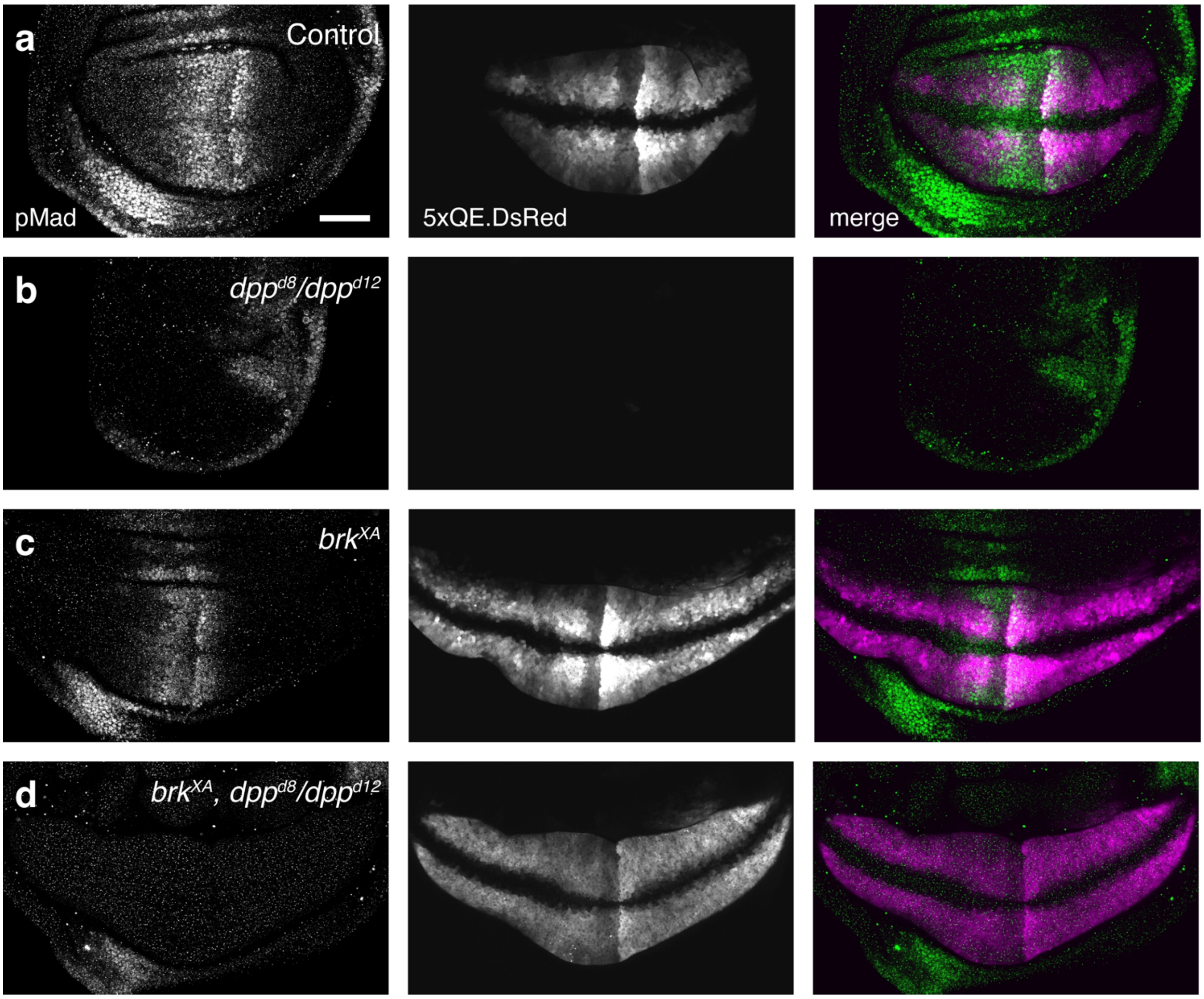


**Supplementary Fig 7. *5xQE.DsRed* reporter expression is largely independent of Dpp signaling**
**a**-**d**, α-pMad, 5xQE.DsRed, and merge of control (**a**), $dpp^{d8}/dpp^{d12}$ (**b**), $brk^{XA}$ (**c**), and $brk^{XA}$; $dpp^{d8}/dpp^{d12}$ (**d**) wing discs. Scale bar 50 µm.

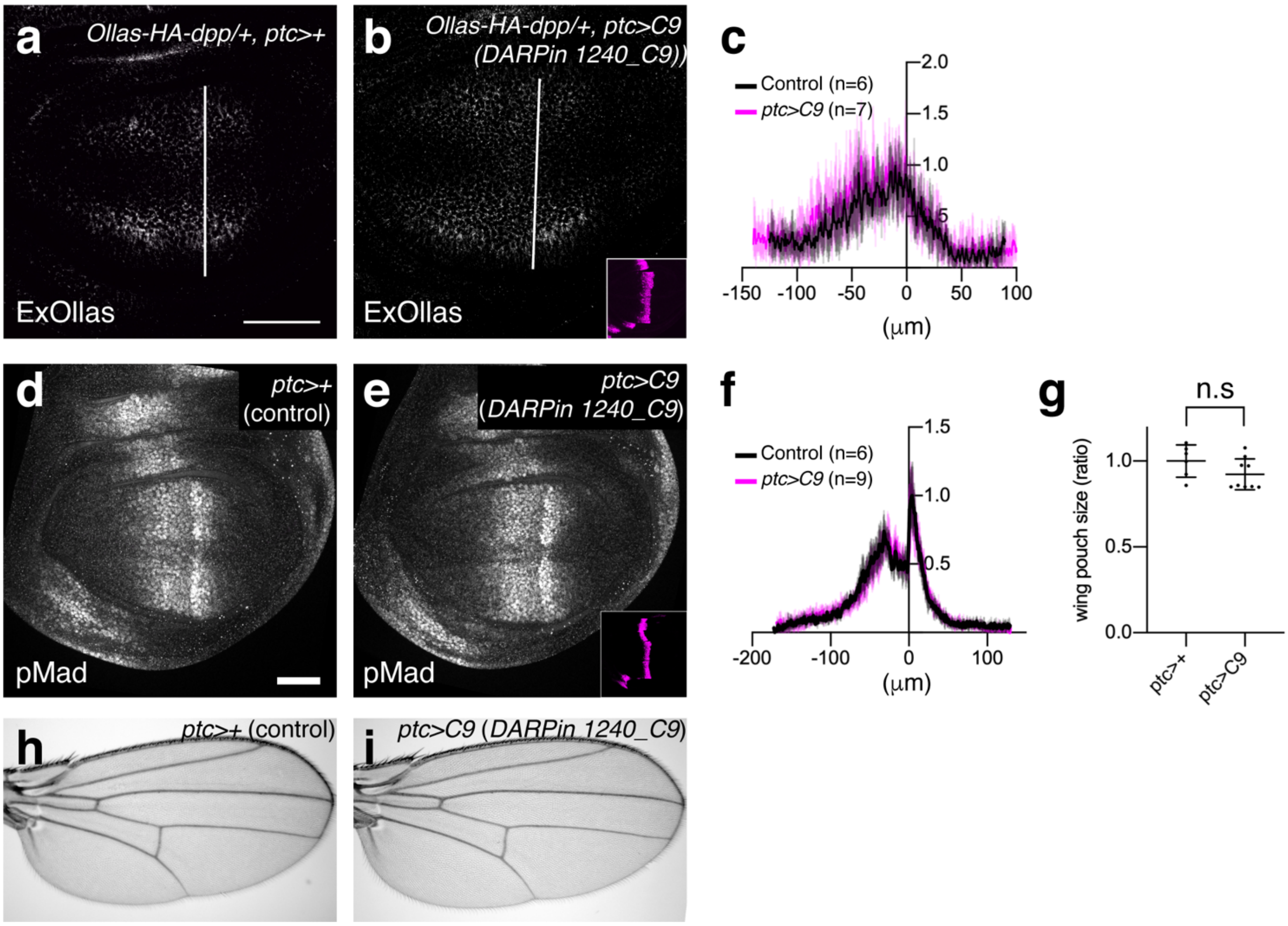


**Supplementary Fig 8. Expression of a trap (containing DARPin 1240_C9) using *ptc*-Gal4 did not affect extracellular distribution of Dpp, pMad signaling, or patterning and growth of the adult wing**

**a-b**, Extracellular α-Ollas staining (ExOllas) of *Ollas-HA-dpp/+, ptc>+* wing disc (control) (**a**), and *Ollas-HA-dpp/+, ptc>C9* wing disc (**b**). **c**, Average fluorescence intensity profile of extracellular α-Ollas staining of (**a-b**). *Ollas-HA-dpp/+, ptc>+* wing disc (control) (*n*=6), and *Ollas-HA-dpp/+, ptc>C9* wing disc (*n*=7). Data are presented as mean+/-SD. **d-e**, α-pMad of *ptc>+* wing disc (control) (**d**), and *ptc>C9* wing disc (**e**). **f**, Average fluorescence intensity profile of α-pMad staining of (**d-e**). *ptc>+* wing disc (control) (*n*=6), and *ptc>C9* wing disc (*n*=9). Data are presented as mean+/-SD. **g**, Comparison of wing pouch size of (**d-e**). *ptc>+* (control) (*n*=6), and *ptc>C9* disc (*n*=9). Data are presented as mean+/-SD. Two-sided Mann-Whitney test was used for comparison (*p*=0.0879). (n.s; not significant). **h-i**, Adult wing of *ptc>+* wing disc (control) (**h**), and *ptc>C9* (**i**). Scale bar 50 μm.

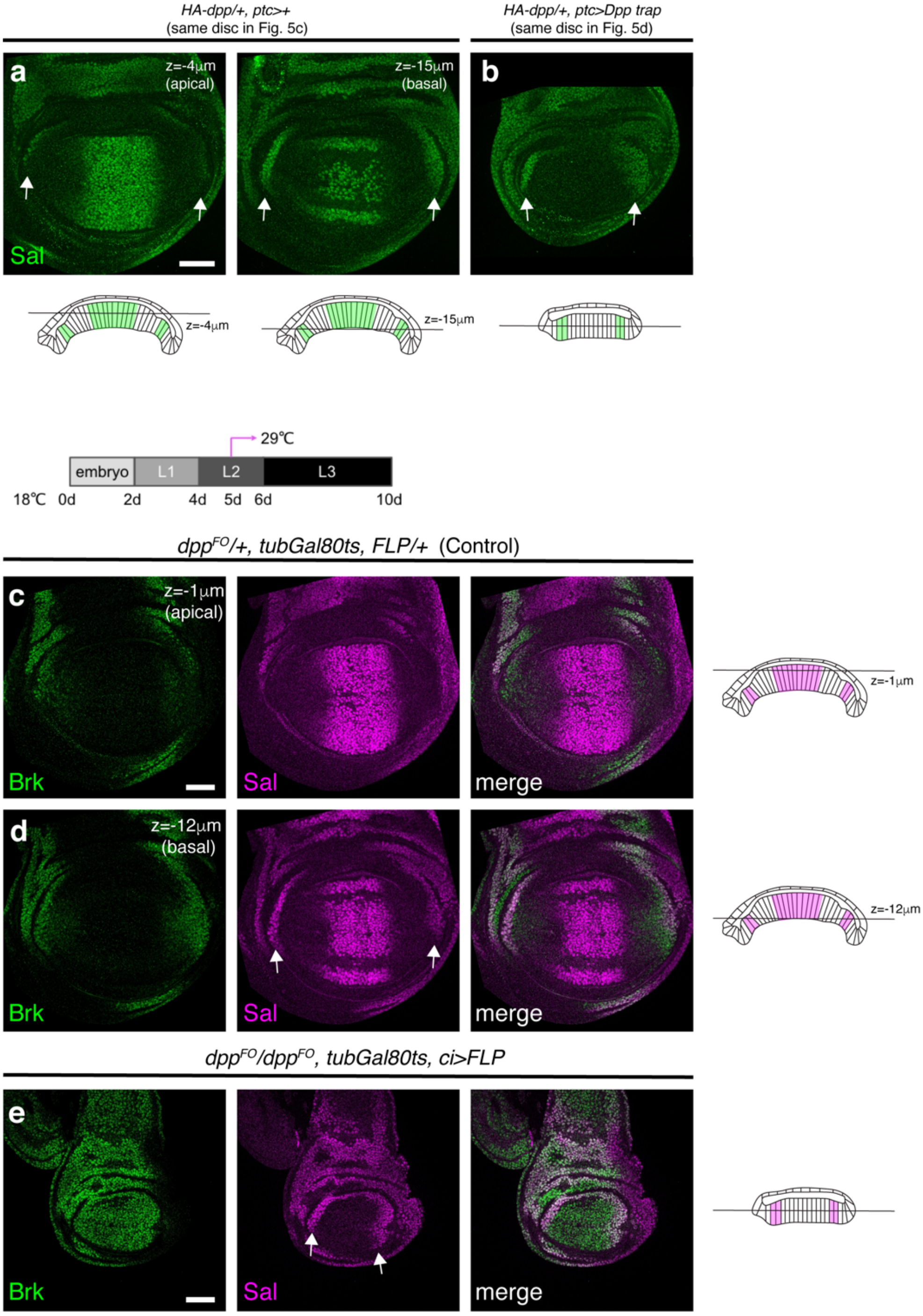


**Supplementary Fig 9. Lateral Sal expression is not affected by loss of Dpp signaling**

**a**-**b**, α-Sal staining of *HA-dpp/+, ptc>+* disc (control) (**a**), and *HA-dpp/+, ptc>Dpp trap* disc (**b**). Each wing disc is from Fig. 5c and Fig. 5d, respectively. In an apical confocal section of control wing disc (z=-4μm), the lateral Sal expression is hidden due to the tissue architecture but in a basal confocal section of control wing disc (z=-15μm), the lateral Sal expression is easily detected (**a**). **c**-**e**, α-Brk, α-Sal, and merge of *dpp*$^{FO}$*/+, tubGal80ts, FLP/+* disc (control) (**c**-**d,** same wing disc), and *dpp*$^{FO}$*/ dpp*$^{FO}$*, tubGal80ts, ci>FLP* disc (**e**). *dpp* was genetically removed from the mid-second instar. The lateral Sal expression is found in a basal confocal section of control wing disc (z=-12μm) (**c**-**d**). The lateral Sal expression is not significantly upregulated, although Brk is uniformly upregulated upon generic removal of *dpp* from the entire A compartment using *ci*-Gal4 (**e**). Scale bar 50 μm.

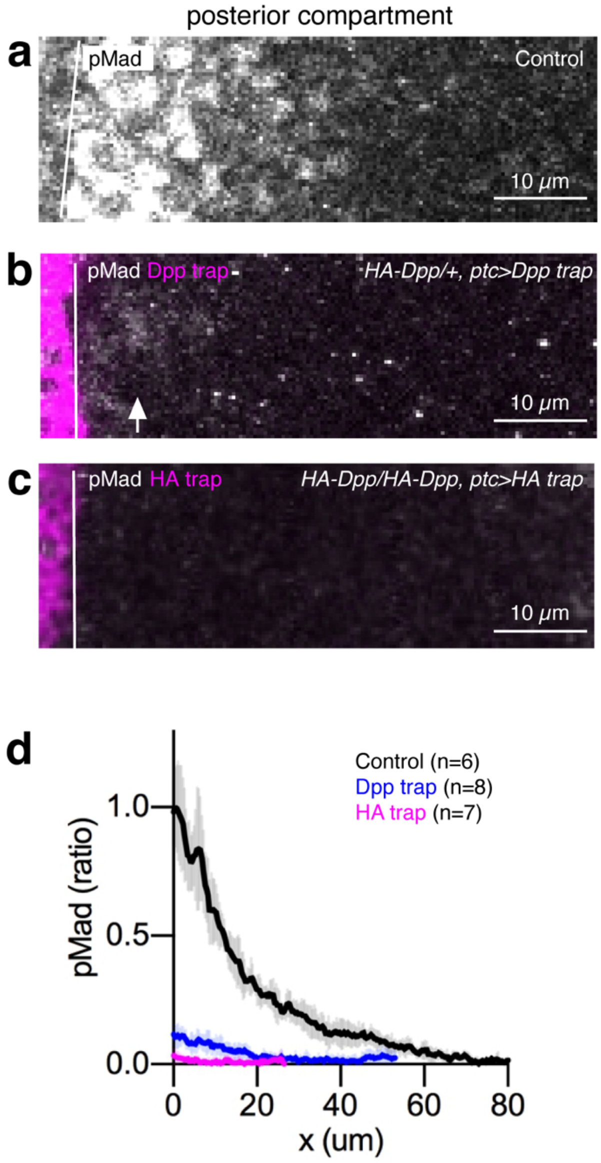


**Supplementary Fig. 10. HA trap can trap Dpp more efficiently than Dpp trap**

**a**-**c**, α-pMad and mCherry (Dpp trap or HA trap) in the P compartment of control wing disc (**a**), *HA-dpp/+, ptc>Dpp trap* wing disc (**b**), and *HA-dpp/HA-dpp, ptc>HA trap* (**c**). Scale bar 10 µm. **d**, Average fluorescence intensity profile of posterior α-pMad staining of (**a**-**c**). Data are presented as mean+/-SD. Arrow indicates pMad signal by leaked Dpp from Dpp trap.

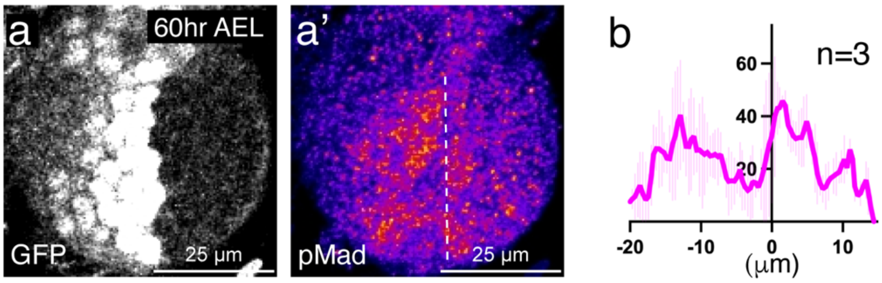


**Supplementary Fig. 11. pMad signaling at mid-second instar stage**
**a-a'**, α-GFP (**a**) and α-pMad (**a'**) staining of wing disc expressing the *d2GFP* reporter at mid-second instar stage (60hr AEL). Dotted line indicates A-P compartment boundary. Scale bar 25 µm. **b**, Average fluorescence intensity profile of α-pMad staining of at mid-second instar stage (60hr AEL) (*n*=3). Data are presented as mean+/-SD.

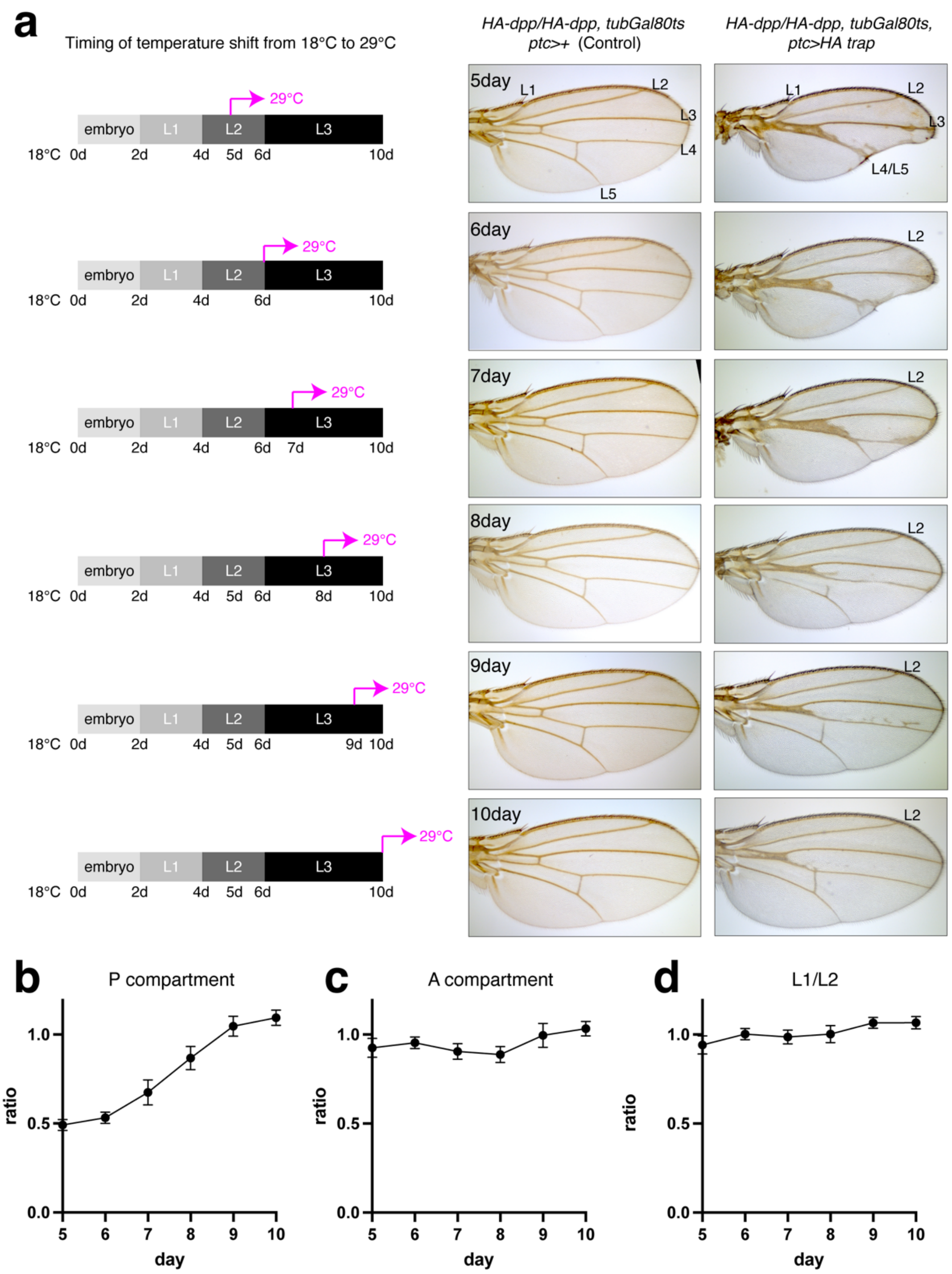


**Supplementary Fig. 12 Relatively normal anterior patterning and growth by blocking Dpp dispersal at different time points**

**a**, *HA-dpp/HA-dpp, tubGal80ts, ptc>+* control adult wings and *HA-dpp/HA-dpp, tubGal80ts, ptc>HA trap* adult wings. Crosses were shifted from 18 °C to 29 °C at indicated time point. **b-d**, P compartment size (**b**), A compartment size (**c**), and the size of the peripheral region between L1 and L2 (**d**) of *HA-dpp/HA-dpp, tubGal80ts, ptc>HA trap* adult wings (*n*=16, 13, 14, 24, 16, 13 at 5, 6, 7, 8, 9, 10 day) were normalized against each counter part size of *HA-dpp/HA-dpp, tubGal80ts, ptc>+* control adult wings (*n*=12, 11, 14, 13, 14, 12 at 5, 6, 7, 8, 9, 10 day). Crosses were shifted from 18 °C to 29 °C at indicated time point. Data are presented as mean+/-SD.

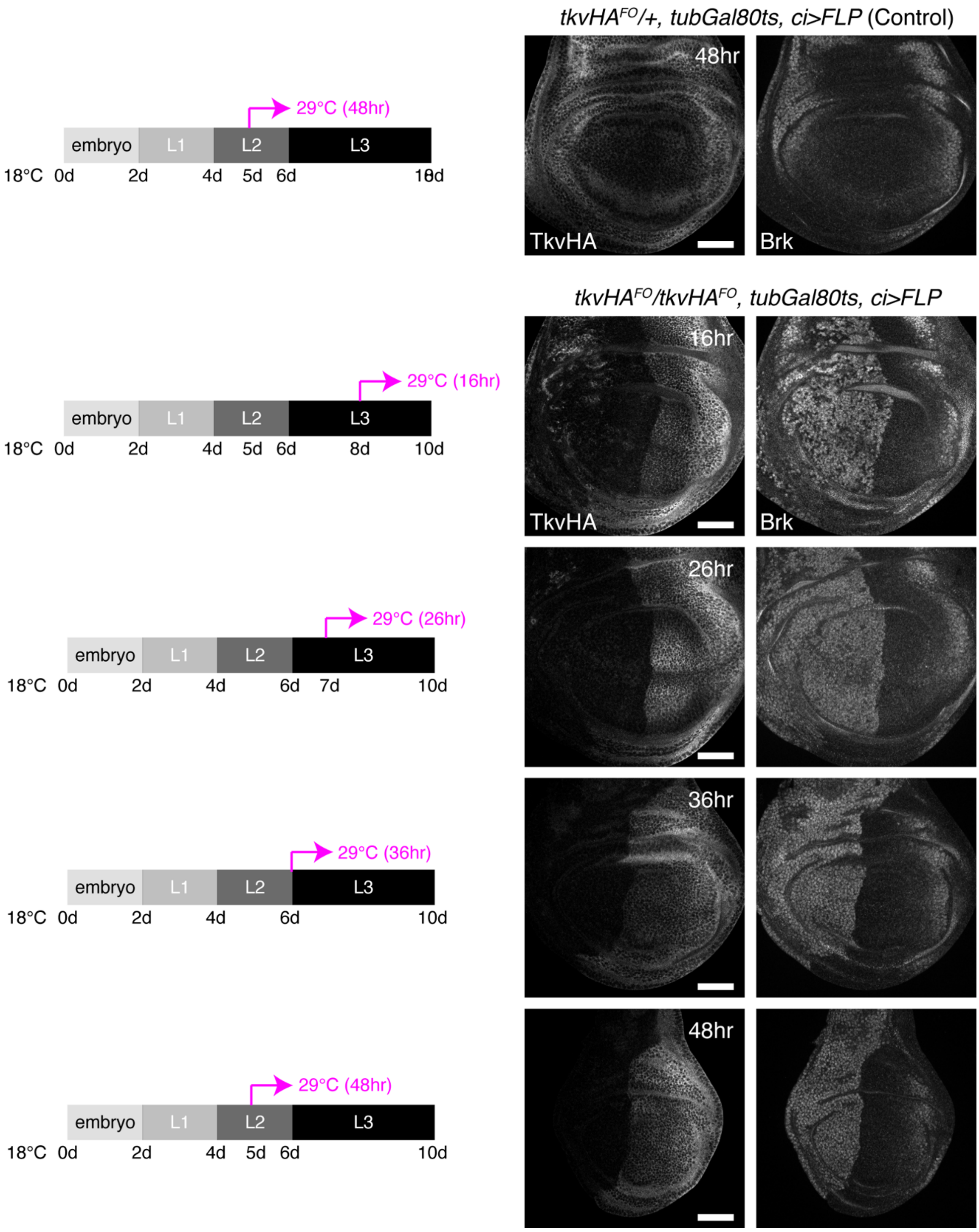


**Supplementary Fig 13. De-repression of Brk by genetic removal of *tkv***

α-HA (TkvHA$^{FO}$) and α-Brk staining of wing discs in which *tkv* was genetically removed from A compartment using *ci*-Gal4 from different time points. The larvae were raised at 18 °C until a temperature shift to 29 °C to induce Gal4 expression. Time shown in each figure indicates the time of dissection after temperature shift. All the discs are the same age and only difference is how long *tkv* had been removed. Scale bar 50 µm.

## SUPPLEMENTARY TABLE 1

### Genotypes by figures

| |
|---|
| Fig. 1c-e: *yw; HA-dpp/HA-dpp* |
| Fig. 2b: *yw; ptc-Gal4, Ollas-HA-dpp/+* |
| Fig. 2d: *yw; ptc-Gal4, Ollas-HA-dpp/+; UAS/LexAop-HA trap/+* |
| Fig. 2f, h: *hsFLP; Ollas-HA-dpp/tub>CD2, Stop>Gal4, UAS-nlacZ; UAS/LexAop-HA trap/+* |
| Fig. 2j: *hsFLP; ptc-Gal4, Ollas-HA-dpp/tub>CD2, Stop>Gal4, UAS-nlacZ; UAS/LexAop-HA trap/+* |
| Fig. 2l: *hsFLP; Ollas-HA-dpp/tub>CD2, Stop>Gal4, UAS-nlacZ; UAS/LexAop-HA trap/+* |
| Fig. 3a, h: (*5xQE.DsRed); ptc-Gal4, HA-dpp/HA-dpp* |
| Fig. 3b, i: (*5xQE.DsRed); ptc-Gal4, HA-dpp/HA-dpp; UAS/LexAop-HA trap/+* |
| Fig. 3k, r: *yw; nub-Gal4, HA-dpp/HA-dpp* |
| Fig. 3l, s: *yw; nub-Gal4, HA-dpp/HA-dpp; UAS/LexAop-HA trap/+* |
| Fig. 4a: *hsFLP/5xQE.DsRed; HA-dpp,* $tkv^{a12}$ *FRT40/HA-dpp, UbiGFP, FRT40, ptc-Gal4; UAS/LexAop-HA trap/+* |
| Fig. 4b: *hsFLP/5xQE.DsRed;* $tkv^{a12}$ *FRT40/UbiGFP, FRT40* |
| Fig. 4c, d: *hsFLP/5xQE.DsRed;* $tkvHA^{FO}/tkvHA^{FO}$ |
| Fig. 4e, f: (internal control within a cross) *5xQE.DsRed/+; (*$dpp^{FO}$*, ci-Gal4)/(*$dpp^{FO}$*); (UAS-FLP)/tubGal80ts,* |
| Fig. 4g, h: *5xQE.DsRed/+;* $dpp^{FO}$*, ci-Gal4/*$dpp^{FO}$*; UAS-FLP/tubGal80ts* |
| Fig. 5b: (left) *yw; ptc-Gal4, Ollas-HA-dpp/+*, (right) *yw; ptc-Gal4, Ollas-HA-dpp/+; UAS/LexAop-Dpp trap/+* |
| Fig. 5c: *yw; ptc-Gal4, HA-dpp/+* |
| Fig. 5d: *yw; ptc-Gal4, HA-dpp/+; UAS/LexAop-Dpp trap/+* |
| Fig. 5k, r: *yw; nub-Gal4, HA-dpp/+* |
| Fig. 5l, s: *yw; nub-Gal4, HA-dpp/+; UAS/LexAop-Dpp trap/+* |
| Fig. 6a, c: (*y*)*w;* (*5xQE.DsRed*)*;* $dpp^{d8}/dpp^{d12}$ |
| Fig. 6b, d: (*y*)*w;* (*5xQE.DsRed*)*;* $dpp^{d8}/dpp^{d12}$*; dpp-Gal4/UAS-tkvQD* |
| Fig. 6f: (*5xQE.DsRed*)*;* $dpp^{d8}$*, UAS-FLP/*$dpp^{d12}$*, act>Stop, y+>*$LexA^{LHG}$*; dpp-Gal4/LexAop-tkvQD* |
| Fig. 7c: *yw; dpp-T2A-Gal4, Dp(2;2)DTD48(dpp+)/+; P{w[+mC]=UAS-RedStinger}6, P{w[+mC]=UAS-FLP.Exel}3, P{w[+mC]=Ubi-p63E(FRT.STOP)Stinger}15F2/+* |
| Fig. 7e-h: *yw M{vas-int.Dm}zh-2A; dpp-T2A-d2GFP-NLS/Cyo, P23* |
| Fig. 7i-l: *yw* |
| Fig. 8a-c: *ptc-Gal4,* $dpp^{FO}$*/+; tubGal80ts/UAS-FLP*, *act5C(FRT.polyA)lacZ.nls* |
| Fig. 8d-h: *ptc-Gal4,* $dpp^{FO}/dpp^{FO}$*; tubGal80ts/UAS-FLP*, *act5C(FRT.polyA)lacZ.nls* |
| Fig. 8i-j: *ci-Gal4,* $dpp^{FO}/dpp^{FO}$*; tubGal80ts/UAS-FLP*, *act5C(FRT.polyA)lacZ.nls* |
| |
| Supplementary Fig. 1a, e: *ptc-Gal4/+* |
| Supplementary Fig. 1b, f: *ptc-Gal4/+; UAS/LexAop-HA trap/+* |
| Supplementary Fig. 2a, c, e, g, i: *HA-dpp/HA-dpp, ci*>+ (left) and *HA-dpp/HA-dpp, ci>HA trap* (right) |
| Supplementary Fig. 2k, m, o, q, s: *HA-dpp/+, ci*>+ (left) and *HA-dpp/+, ci>Dpp trap* (right) |
| Supplementary Fig. 3a: *nub-Gal4, ptc-Gal4, HA-dpp/HA-dpp* (control), |
| Supplementary Fig. 3b: *nub-Gal4, ptc-Gal4, HA-dpp/HA-dpp; UAS/LexAop-HA trap/+* |
| Supplementary Fig. 4a: *ptc-Gal4, HA-dpp/HA-dpp* |
| Supplementary Fig. 4b: *ptc-Gal4, HA-dpp/HA-dpp; UAS/LexAop-HA trap/+* |

| |
|---|
| Supplementary Fig. 4c: *nub-Gal4, HA-dpp/+; UAS/LexAop-Dpp trap/+*, |
| Supplementary Fig. 4e: *nub-Gal4, HA-dpp/HA-dpp; UAS/LexAop-HA trap/+*, |
| Supplementary Fig. 4f: *nub-Gal4, HA-dpp/HA-dpp; UAS/LexAop-HA trap/UAS-p35* |
| Supplementary Fig. 4h: *nub-Gal4, HA-dpp/+; UAS/LexAop-Dpp trap/+* |
| Supplementary Fig. 4i: *nub-Gal4, HA-dpp/+; UAS/LexAop-Dpp trap/UAS-p35* |
| Supplementary Fig. 5a, b: (control within the cross) *5xQE.DsRed/+; (tkvHA*$^{FO}$*, ci-Gal4)/(tkvHA*$^{FO}$*); (UAS-FLP)/tubGal80ts* |
| Supplementary Fig. 5c, d: *5xQE.DsRed/+; tkvHA*$^{FO}$*, ci-Gal4/tkvHA*$^{FO}$*; UAS-FLP/tubGal80ts* |
| Supplementary Fig. 5e, f: (control within the cross) *5xQE.DsRed/+; (tkvHA*$^{FO}$*)/(tkvHA*$^{FO}$*); +/Hh-Gal4, tubGal80ts* |
| Supplementary Fig. 5g, h: *5xQE.DsRed/+; tkvHA*$^{FO}$*/tkvHA*$^{FO}$*; UAS-FLP/Hh-Gal4, tubGal80ts* |
| Supplementary Fig. 6a: (control within the cross) *5xQE.DsRed/+; (tkvHA*$^{FO}$*)/tkvHA*$^{FO}$*; (Hh-Gal4)/+* |
| Supplementary Fig. 6b: (experiment) *5xQE.DsRed/+; tkvHA*$^{FO}$*/tkvHA*$^{FO}$*; Hh-Gal4/UAS-FLP* |
| Supplementary Fig. 7a: *5xQE.DsRed/+, dpp*$^{d8}$ *or dpp*$^{d12}$*/+* |
| Supplementary Fig. 7b: *5xQE.DsRed/+; dpp*$^{d8}$*/dpp*$^{d12}$ |
| Supplementary Fig. 7c: *5xQE.DsRed, brk*$^{XA}$*/Y, dpp*$^{d8}$ *or dpp*$^{d12}$*/+* |
| Supplementary Fig. 7d: *5xQE.DsRed, brk*$^{XA}$*/Y, dpp*$^{d8}$*/dpp*$^{d12}$ |
| Supplementary Fig. 8a: *Ollas-HA-dpp, ptc-Gal4/+* |
| Supplementary Fig. 8b: *Ollas-HA-dpp, ptc-Gal4/+; UAS/LexAop-C9/+* |
| Supplementary Fig. 8d, h: *ptc-Gal4/+* |
| Supplementary Fig. 8e, i: *ptc-Gal4/+; UAS/LexAop-C9/+* |
| Supplementary Fig. 9a: *yw; ptc-Gal4, HA-dpp/+* (identical disc as Fig. 5c) |
| Supplementary Fig. 9b: *yw; ptc-Gal4, HA-dpp/+; UAS/LexAop-Dpp trap/+* (identical disc as Fig. 5d) |
| Supplementary Fig. 9c, d: *dpp*$^{FO}$*/+; UAS-FLP/tubGal80ts* |
| Supplementary Fig. 9e: *dpp*$^{FO}$*, ci-Gal4/dpp*$^{FO}$*; UAS-FLP/tubGal80ts* |
| Supplementary Fig. 10a: (control) *ptc-Gal4, HA-dpp/+*, |
| Supplementary Fig. 10b: (Dpp trap) *ptc-Gal4, HA-dpp/+; UAS/LexAop-Dpp trap/+* |
| Supplementary Fig. 10c: (HA trap) *ptc-Gal4, HA-dpp/HA-dpp; UAS/LexAop-HA trap/+* |
| Supplementary Fig. 11: *yw M{vas-int.Dm}zh-2A; dpp-T2A-d2GFP-NLS/Cyo, P23* |
| Supplementary Fig. 12: (control) *ptc-Gal4, HA-dpp/HA-dpp; tubGal80ts/+*, (experiment) *ptc-Gal4, HA-dpp/HA-dpp; UAS/LexAop-HA trap/tubGal80ts* |
| Supplementary Fig. 13: (control) *tkvHA*$^{FO}$*/+; UAS-FLP/tubGal80ts*, (experiment) *tkvHA*$^{FO}$*, ci-Gal4/tkvHA*$^{FO}$*; UAS-FLP/tubGal80ts* |

## SUPPLEMENTARY TABLE 2

Primer lists to determine the orientation of the *dpp* genomic fragment insertion.

| | | |
|---|---|---|
| S25 | mCherry-AgeI-F | CCACCGGTCGCCACCATGGTGAGCAAGGGCGA |
| S85 | Dpp mimic-F1 | GCGGCCGCCCAAGATCGACCGCTCC |
| S86 | Dpp mimic-R1 | CGCGGTGCACAAAAGCCTAGGCGGATGGC |

S25/S86 for the right orientation.
S25/S85 for the wrong orientation.
