# Supplementary material for: Asymmetric requirement of Dpp/BMP morphogen dispersal in the Drosophila wing disc
Source: Nat Commun. 2021 Nov 8;12:6435. doi: 10.1038/s41467-021-26726-6 (PMC8576045; doi:10.1038/s41467-021-26726-6)
Supplement: Supplementary file 3 — Reporting Summary [file 41467_2021_26726_MOESM3_ESM.pdf]

nature research

Corresponding author(s): Shinya Matsuda and Markus Affolter

Last updated by author(s): Oct/10/21

Reporting Summary

Nature Research wishes to improve the reproducibility of the work that we publish. This form provides structure for consistency and transparency in reporting. For further information on Nature Research policies, see our Editorial Policies and the Editorial Policy Checklist.

Statistics

For all statistical analyses, confirm that the following items are present in the figure legend, table legend, main text, or Methods section.

n/a	Confirmed	
☐	☒	The exact sample size (*n*) for each experimental group/condition, given as a discrete number and unit of measurement
☐	☒	A statement on whether measurements were taken from distinct samples or whether the same sample was measured repeatedly
☐	☒	The statistical test(s) used AND whether they are one- or two-sided<br>*Only common tests should be described solely by name; describe more complex techniques in the Methods section.*
☒	☐	A description of all covariates tested
☒	☐	A description of any assumptions or corrections, such as tests of normality and adjustment for multiple comparisons
☐	☒	A full description of the statistical parameters including central tendency (e.g. means) or other basic estimates (e.g. regression coefficient) AND variation (e.g. standard deviation) or associated estimates of uncertainty (e.g. confidence intervals)
☐	☒	For null hypothesis testing, the test statistic (e.g. *F*, *t*, *r*) with confidence intervals, effect sizes, degrees of freedom and *P* value noted<br>*Give P values as exact values whenever suitable.*
☒	☐	For Bayesian analysis, information on the choice of priors and Markov chain Monte Carlo settings
☒	☐	For hierarchical and complex designs, identification of the appropriate level for tests and full reporting of outcomes
☒	☐	Estimates of effect sizes (e.g. Cohen's *d*, Pearson's *r*), indicating how they were calculated

Our web collection on statistics for biologists contains articles on many of the points above.

Software and code

Policy information about availability of computer code

Data collection	Leica SP5-II-MATRIX and Leica LAS AF (ver. 2.6.0.7266)-confocal image acquisition<br>wing_disc-alignment.py -- custom code for generating average signal intensity profile after alignmentof the profile along the A/P compartment boundary.<br>wingdisc_comparison.py -- custom code for comparing control and experimental average intensity profile<br>Two programs can be obtained from https://etiennees.github.io/Wing_disc-alignment/
Data analysis	ImageJ (v.2.0.0-rc69/1.52p)-image analysis<br>Illustrator (24.1.3)-figure preparation<br>Prism (v.8.4.3(471))-graph preparation and statistical test calculation<br>Omero(ver 5.9.1)-figure preparation<br>Excel (Ver. 16.51)-data analysis

For manuscripts utilizing custom algorithms or software that are central to the research but not yet described in published literature, software must be made available to editors and reviewers. We strongly encourage code deposition in a community repository (e.g. GitHub). See the Nature Research guidelines for submitting code & software for further information.

Data

Policy information about availability of data

All manuscripts must include a data availability statement. This statement should provide the following information, where applicable:

- Accession codes, unique identifiers, or web links for publicly available datasets
- A list of figures that have associated raw data
- A description of any restrictions on data availability

The accession numbers for anti-HA antibody is (VH: 1-423 of LC522514 and VL: 67-420 of LC522515).
The accession numbers for the probes against dpp target 682-1673 (NM_057963.5)

Field-specific reporting

Please select the one below that is the best fit for your research. If you are not sure, read the appropriate sections before making your selection.

☒ Life sciences ☐ Behavioural & social sciences ☐ Ecological, evolutionary & environmental sciences

For a reference copy of the document with all sections, see nature.com/documents/nr-reporting-summary-flat.pdf

Life sciences study design

All studies must disclose on these points even when the disclosure is negative.

Sample size	The sample sizes were not predetermined by any statistical methods, but estimated following a previous work (Harmansa et al., 2015) to ensure statistical significance.
Data exclusions	No data were excluded except if technical reason applied such as damaging samples during dissections and preparations of samples or staining failure.
Replication	Experiments were performed at least three independent biological replicates. The experiments were repeated at least two times independently with similar results. All attempts were successful.
Randomization	Randomization was not used, since all the experiments were categorized by distinct genotypes.
Blinding	Blinding was not possible since the phenotypes differ strikingly from controls and the experimental setup is easily identified from control.

Reporting for specific materials, systems and methods

We require information from authors about some types of materials, experimental systems and methods used in many studies. Here, indicate whether each material, system or method listed is relevant to your study. If you are not sure if a list item applies to your research, read the appropriate section before selecting a response.

Materials & experimental systems

n/a	Involved in the study	
☐	☒	Antibodies
☒	☐	Eukaryotic cell lines
☒	☐	Palaeontology and archaeology
☐	☒	Animals and other organisms
☒	☐	Human research participants
☒	☐	Clinical data
☒	☐	Dual use research of concern

Methods

n/a	Involved in the study	
☒	☐	ChIP-seq
☒	☐	Flow cytometry
☒	☐	MRI-based neuroimaging

Antibodies

Antibodies used	The following primary antibodies were used in this study. anti-HA (3F10, Roche 11867423001, RRID:AB_390914), anti-Ollas (L2, Novus Biologicals, NBP1-06713, RRID:AB_1968650), anti-phospho-Smad1/5 (41D10, Cell Signaling, #9516), anti-Brk (generated by Gines Morata), anti-Sal (generated Rosa Barrio), anti-Omb (generated by Gert Pflugfelder), anti-Wg (DSHB, University of Iowa, AB_528512), anti-Ptc (DSHB, University of Iowa, AB_528441), anti-β-Galactosidase (Z3781, Promega, AB_430877), anti-β-Galactosidase (ab9361, abcam, AB_307210), and anti-Cleaved Caspase-3 (#9661, Cell Signaling). The following secondary antibodies were used in this study. Goat anti-Chicken IgY (H+L) DyLight 680(#SA5-10074, Invitrogen), Alexa Fluor® 488 AffiniPure Goat Anti-Mouse IgG, Fcγ fragment specific (115-545-071, Jackson ImmunoResearch), Goat anti-Mouse IgG (H+L) Alexa Fluor Plus 488 (A32723, Thermo Fisher), Goat anti-Mouse IgG (H+L) Alexa Fluor 568 (#A-11004, Thermo Fisher), Alexa Fluor® 680 AffiniPure Goat Anti-Mouse IgG, Fcγ fragment specific (115-625-071, Jackson ImmunoResearch), Goat anti-Rabbit IgG (H+L) Alexa Fluor 488 (#A-11008, Thermo Fisher), F(ab')2-Goat anti-Rabbit IgG (H+L) Alexa Fluor 568(#A-21069, Thermo Fisher), Goat anti-Rabbit IgG (H+L) Alexa Fluor 680 (#

	A-21109, Thermo Fisher), Goat anti-Guinea Pig IgG (H+L) Alexa Fluor 488 (#A-11073, Thermo Fisher), Goat Anti-Rat IgG Fc (FITC) (ab97089, Abcam), Goat anti-Rat IgG (H+L), Alexa Fluor 680 (#A-21096, Thermo Fisher).
Validation	All the primary antibodies listed here were validated for immunostaining of the Drosophila wing disc. anti-HA (PMID: 20453847), anti-Ollas (PMID: 23035643), anti-phospho-Smad1/5 (PMID: 26550827), anti-Brk (PMID: 26550827), anti-Sal (PMID: 26550827), anti-Omb (PMID: 26550827), anti-Wg (PMID: 26550827), anti-Ptc (PMID: 26550827), anti-β-Galactosidase (Z3781, PMID: 26550824), anti-β-Galactosidase (ab9361, PMID: 30718556), anti-Cleaved Caspase-3 (PMID: 32633716).

Animals and other organisms

Policy information about studies involving animals; ARRIVE guidelines recommended for reporting animal research

Laboratory animals	Drosophila melanogaster. Males and Females. Details of the lines generated and used in this study can be found in the methods.
Wild animals	The study did not involve wild animals.
Field-collected samples	The study did not involve field-collected samples.
Ethics oversight	No ethical approval or guidance required for invertebrate model animals like Drosophila.

Note that full information on the approval of the study protocol must also be provided in the manuscript.
